# MyD88 Signaling Accompanied by Microbiota Changes Supports Urinary Bladder Carcinogenesis

**DOI:** 10.3390/ijms25137176

**Published:** 2024-06-29

**Authors:** Dora Knezović, Blanka Milić Roje, Katarina Vilović, Lucija Franković, Jelena Korac-Prlic, Janoš Terzić

**Affiliations:** 1 Laboratory for Cancer Research, University of Split School of Medicine, Šoltanska 2A, 21000 Split, Croatia; dora.knezovic@mefst.hr (D.K.); blanka.roje@mefst.hr (B.M.R.); lucija.frankovic@mefst.hr (L.F.); jelena.korac.prlic@mefst.hr (J.K.-P.); 2 Department of Pathology, Forensic Medicine and Cytology, University Hospital of Split, Spinčićeva 1, 21000 Split, Croatia; katarina.vilovic@mefst.hr

**Keywords:** urinary bladder neoplasms, myeloid differentiation factor 88, Toll-like receptor 4, microbiota, *Faecalibaculum*

## Abstract

Urinary bladder cancer (BC) inflicts a significant impairment of life quality and poses a high mortality risk. *Schistosoma haematobium* infection can cause BC, and the urinary microbiota of BC patients differs from healthy controls. Importantly, intravesical instillation of the bacterium *Bacillus Calmette-Guerin* stands as the foremost therapy for non-muscle invasive BC. Hence, studying the receptors and signaling molecules orchestrating bacterial recognition and the cellular response in the context of BC is of paramount importance. Thus, we challenged Toll-like receptor 4 (*Tlr4*) and myeloid differentiation factor 88 (*Myd88*) knock-out (KO) mice with N-butyl-N-(4-hydroxylbutyl)-nitrosamine (BBN), a well-known urinary bladder carcinogen. Gut microbiota, gene expression, and urinary bladder pathology were followed. Acute exposure to BBN did not reveal a difference in bladder pathology despite differences in the animal’s ability to recognize and react to bacteria. However, chronic treatment resulted in reduced cancer invasiveness among *Myd88*^KO^ mice while the absence of functional *Tlr4* did not influence BC development or progression. These differences correlate with a heightened abundance of the *Faecalibaculum* genus and the lowest microbial diversity observed among *Myd88*^KO^ mice. The presented data underscore the important role of microbiota composition and MyD88-mediated signaling during bladder carcinogenesis.

## 1. Introduction

Bladder cancer (BC) is one of the most prevalent cancers with 573,278 new cases globally as reported in 2020, thus representing a major health issue worldwide [1]. Men are four times more likely to develop BC, while women tend to experience higher grade and stage tumors, and consequently, a higher risk of progression and recurrence [2]. Based on bladder wall invasion status, BC is categorized into two groups. Non-muscle-invasive bladder cancer (NMIBC) accounts for 80% of BC cases. The prognosis for NMIBC is relatively good with high 5-year survival rates; however, the recurrence rate of 60–70% is still one of the highest compared to other malignancies [3,4]. Muscle-invasive bladder cancer (MIBC) is more aggressive with approximately half of the patients ultimately developing micrometastases [5], thus requiring a more radical therapeutic approach such as cystectomy or trimodal therapy [6]. It is well recognized that, besides smoking and occupational exposure in the paint, rubber, or aluminum industries, chronic inflammation caused by uropathogenic *Escherichia coli* and *Schistosoma haematobium* infections present major risk factors and are associated with the development of BC [1,6,7,8]. Furthermore, differences in urinary microbiota composition were described between BC patients and healthy volunteers [9]. Not only can microbes influence BC development, but they are also a key medication for bladder cancer. The gold standard for treating high-grade NMIBC is a combination of surgical transurethral resection of the bladder tumor (TURBT) followed by the instillation of the *Bacillus Calmette–Guérin* (BCG) bacterium [6]. Following the development of next-generation sequencing methods [10], new insight into BC tissue and urine microbiomes opened a new era of BC research [9,11]. Therefore, studies questioning the role of Toll-like receptors (TLRs), receptors responsible for sensing microbial presence, are becoming highly important. Analyzing the contribution of molecules that are transducing signals of microbial presence to the cell interior [12] can give us fundamental insight into the importance of bacteria in cancer development [13,14]. TLRs are, besides being expressed by immune cells, also present in normal urothelium and BC cells [15]. TLR4, the predominant TLR in the urothelium [15], responds to LPS from Gram-negative bacteria, activating two possible downstream signaling pathways, the myeloid differentiation factor 88 (MyD88)-dependent and TRIF-dependent pathways [16]. MyD88 is an important downstream signaling molecule for all other TLRs, except for TLR3. Present data suggests that both TLR4 and MyD88 have been involved in the progression of multiple cancers, but they also demonstrate a protective role when stimulated or inhibited depending on the cancer type or stage; thus, two TLR4 ligands have already been approved for cancer treatment [17]. Considering the complex interplay of these molecules in carcinogenesis and the need to develop new treatments, it is necessary to obtain a further understanding of their role in bladder carcinogenesis. Not only may the microbiota present a reservoir for possible pathogens, but it might also influence protective responses in other tissues, including the urinary bladder [18,19]. This study aimed to investigate whether the receptor TLR4 and signaling molecule MyD88 have a role in BC development, in the context of parallel gut microbiota and gene expression changes. As the molecules responsible for recognizing and responding to microorganisms, TLR4 and MyD88 were expected to trigger a strong immune response and inflammation, potentially promoting urinary bladder carcinogenesis. Therefore, we hypothesized that the absence of functional TLR4 and MyD88 would confer a certain degree of protection to mice during tumorigenesis.

## 2. Results

### 2.1. Urinary Bladder Pathology Following BBN Exposure

**Figure 1:**
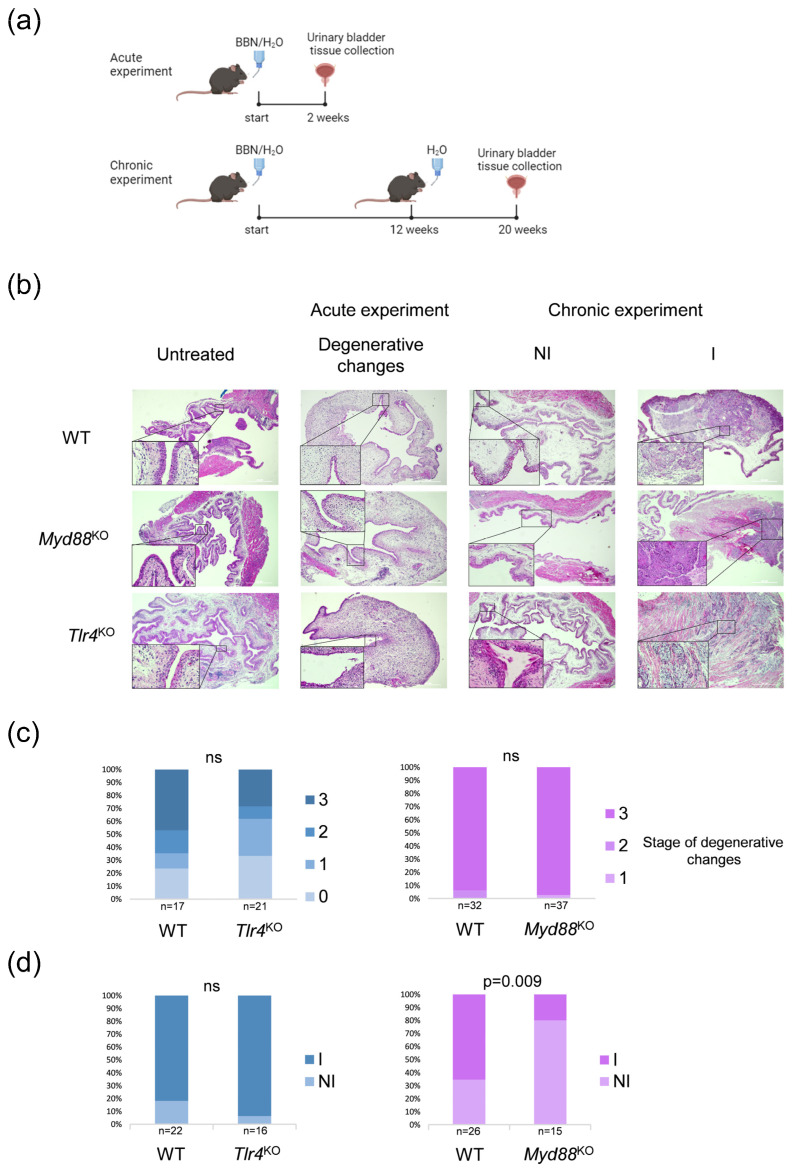
Histopathological assessment of urinary bladder tissue. (**a**) BBN-induced urinary bladder cancer (BC) model for the acute and chronic experiment (created with BioRender); (**b**) Hematoxylin and eosin (H&E) staining of urinary bladder tissue in *Myd88*^KO^, *Tlr4*^KO^, and WT groups (4× and 40× magnification); (**c**) Prevalence of stages of degenerative changes in urinary bladder epithelium in the acute experiment among *Myd88*^KO^, *Tlr4*^KO^, and WT mice groups; (**d**) Prevalence of non-invasive and invasive BC after the chronic experiment in *Myd88*^KO^, *Tlr4*^KO^, and WT mice.; NI—non-invasive; I—invasive; significance tested using Fisher’s exact test; ns—nonsignificant.

Histopathological assessment of urinary bladder tissue was performed following acute and chronic experiments on WT, *Myd88* knock-out (KO), and *Tlr4*^KO^ mice, as well as their corresponding, non-treated controls (Figure 1a,b). Samples from acute experiments were analyzed for the presence of degenerative changes (Stages 1–3) or their absence (Stage 0). In chronic experiments, samples were classified as invasive, if the presence of cancerous cells was detected in the subepithelial connective tissue with or without muscle invasion, or as non-invasive, which means that malignant cells did not invade through the basal membrane. The control groups, undergoing no treatment, revealed normal histological characteristics. An analysis of samples from acute experiments revealed no differences between *Myd88*^KO^ and *Tlr4*^KO^ mice compared with the WT group, although all mice from the *Myd88*^KO^ group had degenerative changes detected, mostly Stage 3 (Figure 1c). Samples from chronic experiments showed no differences between *Tlr4*^KO^ and WT mice regarding bladder cancer invasiveness, meaning that both groups acquired mostly invasive tumors. On the other hand, the *Myd88*^KO^ group developed primarily non-invasive changes, predominantly CIS (n = 5) and dysplasia (n = 4), which is significantly different from the WT group characterized by mostly invasive tumors (Figure 1d). Considering the predominance of non-invasive tumors in the *Myd88*^KO^ group, inflammation was evaluated in both acute and chronic experiments, comparing *Myd88*^KO^ and WT samples. The inflammation scoring system ranged from zero to three, with zero indicating no inflammation, one indicating mild focal inflammation, two indicating moderate focal inflammation, and three indicating severe focal inflammation. There were no significant differences in inflammatory scores between *Myd88*^KO^ and WT samples in either the acute or chronic experiments (*p* = 0.805 and *p* = 0.287, respectively) (Supplementary Figures S1a,b).

### 2.2. Gut microbiota Changes during Bladder Cancer Development

**Figure 2:**
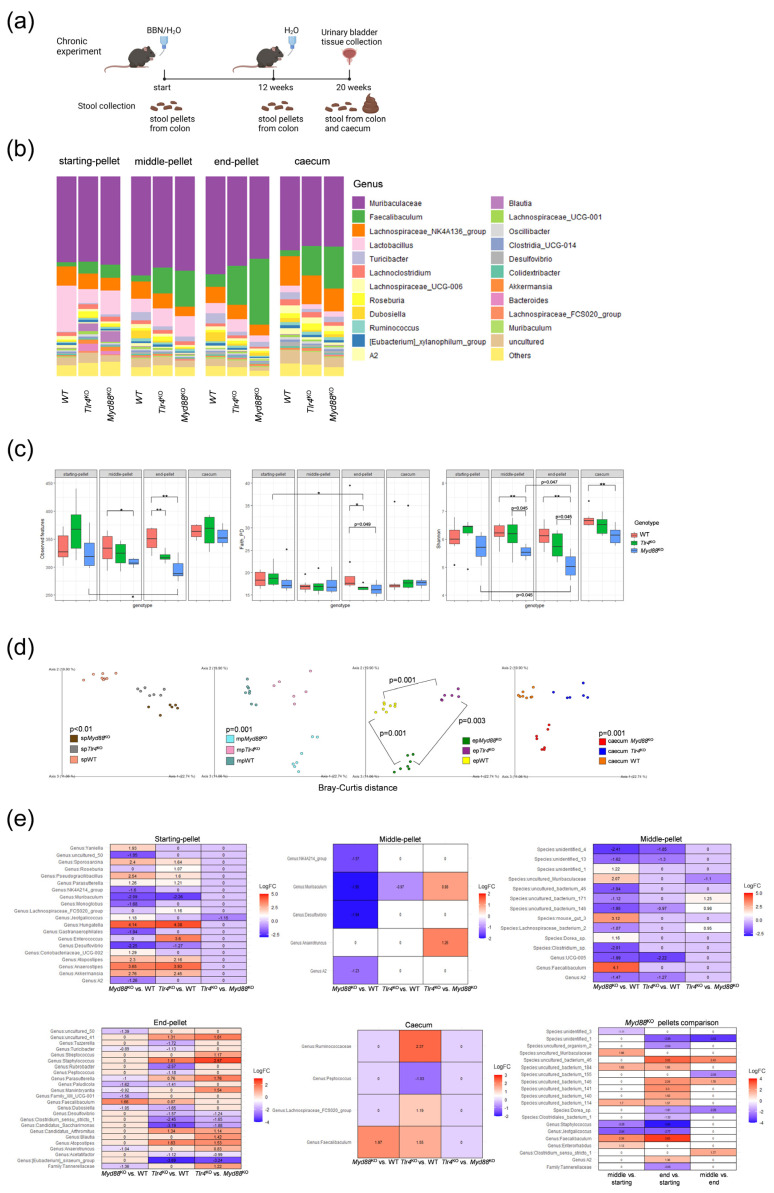
16S metagenomic analysis of stool samples from the chronic experiment. (**a**) Timepoints when stool was collected throughout the chronic experiment in the *Myd88*^KO^, *Tlr4*^KO^, and WT groups (created with BioRender); (**b**) Taxonomy bar plot for genus taxonomic level considering time points and mice groups; (**c**) Alpha diversity measures considering time points and mice groups; (**d**) PCoA plots representing the Bray–Curtis beta diversity measure considering time points and mice groups; (**e**) ANCOM-BC2 differential abundance analysis considering timepoints and mice groups; sp—starting pellet (before the BBN treatment); mp—middle pellet (after 12 weeks of BBN treatment); ep—end pellet (at the end of the chronic experiment after 20 weeks); FC—fold change; * *p* < 0.05; ** *p* < 0.01.

To assess the concomitant microbiota changes during tumor development, 16S metagenomic analysis was performed on DNA from stool samples from *Myd88*^KO^, *Tlr4*^KO^, and WT mice (Figure 2a).

84 samples generated a total of 6,563,005 tags, with an average of 78,131 tags per sample. These tags were assigned to 2584 amplicon sequence variants (ASVs). Supplementary Figures S2a,b show the relative abundance of the predominant bacterial phyla and families, respectively. *Firmicutes* and *Bacteroidota* were the most prevalent phyla, accounting for more than 90% of observed phyla per sample, while *Muribaculaceae*, *Lachnospiraceae*, *Erysipelotrichaceae*, *Lactobacillaceae*, and *Oscillospiraceae* were the five most abundant families detected in all samples. Figure 2b displays the relative abundance of the predominant bacterial genera observed in all samples divided by genotype and the timepoint when the stool was collected during the chronic experiment. The genus *Muribaculaceae* was the most abundant genus, followed by *Faecalibaculum* displaying an increasing trend in relative abundance throughout the chronic experiment within the *Myd88*^KO^ group.

Before the BBN treatment, the tested genotype groups did not differ significantly regarding alpha diversity measures, considering richness, phylogenetic diversity, and evenness (Figure 2c), although the *Myd88*^KO^ group expressed the lowest diversity values. Expectedly, the alpha diversity measures were lower after 12 weeks of BBN treatment (middle pellet), and slightly recovered or remained the same during the following 8 weeks without BBN (end pellet), although some oscillations were not significant. Surprisingly, the values of the observed features (richness) and Shannon index (richness and evenness) dropped significantly (*p* < 0.05) from the beginning until the end of the chronic experiment only in the *Myd88*^KO^ group. All three alpha diversity measures also showed significantly lower indices in the *Myd88*^KO^ group compared to WT after 20 weeks of experiment. In samples collected from the caecum at the end of the chronic experiment, the data showed a drop in richness and evenness in *Myd88*^KO^ mice compared to WT.

Intergroup beta diversity comparison was assessed through the Bray–Curtis (Figure 2d) and weighted UniFrac (Supplementary Figure S2c) measures. Bray–Curtis distance PCoA plots revealed that at each timepoint throughout the chronic experiment, WT, *Tlr4*^KO^, and *Myd88*^KO^ mice cluster significantly, considering richness and evenness. The weighted UniFrac measure, which also takes phylogenetic diversity into account, showed significant separation of groups as well, although segregations on PCoA plots were not obvious (Supplementary Figure S2c).

To explore whether there are differentially abundant taxa between mice groups at different timepoints during the chronic experiment, an ANCOM-BC2 test was performed (Figure 2e). Before the BBN treatment (starting pellet), *Myd88*^KO^ and *Tlr4*^KO^ mice had a more than 17 (*p* < 0.001) and 20 (*p* < 0.001) times higher abundance of the genus *Hungatella* compared to WT group, respectively, followed by a more than 12 times higher abundance of the genus *Anaerostipes* (*p* < 0.001) and a more than 5 times higher abundance of the genus *Akkermansia* (*p* < 0.001). The genera *Muribaculum* and *Desulfovibrio* showed a reduced abundance, more than four times, in the KO groups compared to WT mice (*p* < 0.001). After 12 weeks of BBN treatment (middle pellet), there were fewer differences between groups on the genus level, and they were mostly present in *Myd88*^KO^ mice. The genera *Muribaculum* and *Desulfovibrio* remained less abundant, although to a lesser extent (*p* < 0.001). On a species level, there were more taxa that were less abundant in the *Myd88*^KO^ group compared to WT, but most of them were uncultured. Species related to the genus *Faecalibaculum* turned out to be 17 times more abundant in *Myd88*^KO^ mice compared to WT (*p* < 0.001). At the end of the experiment (end pellet and caecum), following 12 weeks of BBN and 8 weeks of H_2_O treatment, stool from the colon and caecum was collected. The genus *Faecalibaculum* remained more abundant in *Myd88*^KO^ mice compared to WT, three times higher in colon samples (*p* < 0.001) and almost four times in caecum samples (*p* < 0.001). Further examination of the *Myd88*^KO^ group throughout time points (*Myd88*^KO^ pellets’ comparison) revealed a consistent pattern, mirroring the taxonomic distribution illustrated in the taxa bar plot and confirming earlier findings regarding the changes in abundance of the genus *Faecalibaculum*. More precisely, this genus became more abundant after BBN treatment (5 times) until the end of the 20 weeks of the experiment (14 times), compared to the starting point before BBN treatment onset (*p* < 0.001). The genus *Staphylococcus* showed a decreasing abundance, almost 5 times lower after BBN treatment (*p* < 0.001) and 14 times at the end of the experiment (*p* < 0.001) compared to starting point.

### 2.3. Gene Expression Changes among Myd88^KO^ Mice

**Figure 3:**
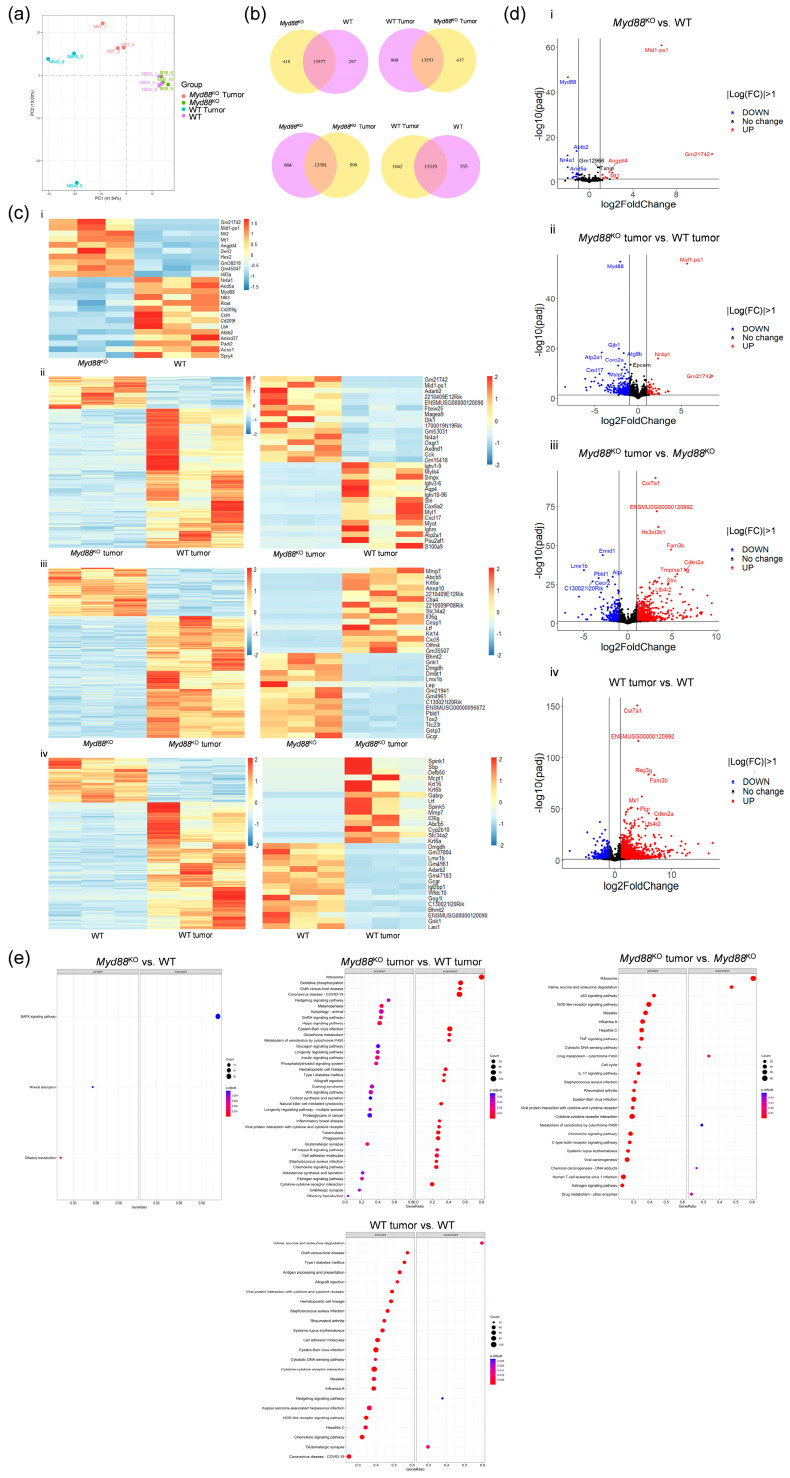
Gene expression profiling of *Myd88*^KO^ untreated bladder tissue and tumors. (**a**) Principal component analysis (PCA) plot showing the grouping of non-treated samples and intergroup differences of *Myd88*^KO^ and WT tumor samples; (**b**) Venn diagrams show the number of uniquely expressed genes within each group and the number of co-expressed genes in both mice groups; (**c**) Heatmaps of significantly differentially expressed genes between mice groups, all genes (left) as well as most prominently changed genes (right) are shown; i—comparison between bladder tissue unexposed to BBN (NT); ii—comparison between *Myd88*^KO^ and WT tumor samples; iii—comparison between *Myd88*^KO^ tumor and non-treated bladder tissue samples; iv—comparison between WT tumor and non-treated bladder tissue samples; (**d**) Volcano plots showing significantly differentially expressed genes between *Myd88*^KO^ and WT tumor and control samples; i—comparison between bladder tissue unexposed to BBN (NT); ii—comparison between *Myd88*^KO^ and WT tumor samples; iii—comparison between *Myd88*^KO^ tumor and non-treated bladder tissue samples; iv—comparison between WT tumor and non-treated bladder tissue samples; (**e**) Pathway enrichment analysis showing the 20 most activated and suppressed KEGG pathways. The size of the dots presents the number of genes annotated to a particular KEGG pathway and the dot color stands for the significance level of the enrichment; FC—fold change.

Given that *Myd88*^KO^ mice had less invasive bladder cancers, it was intriguing to explore the transcriptome profiles of *Myd88*^KO^ and WT non-invasive (NI) tumor samples, along with their respective non-treated (NT) controls. Principal component analysis (PCA) (Figure 3a) demonstrated sample separation between tumor and NT bladder tissue. While the *Myd88*^KO^ and WT non-treated tissue samples did not separate, the tumor samples grouped clearly, although WT NI tumor samples showed a sign of heterogeneity. Figure 3b demonstrates a number of uniquely expressed genes, as well as genes shared between the tested groups.

In bladder tissue unexposed to BBN (NT), only 25 genes were differentially expressed with more than half of them being downregulated in the *Myd88*^KO^ group compared to WT (Figure 3c(i)). When comparing *Myd88*^KO^ and WT tumor samples, 454 genes were differentially expressed, with most of the genes being downregulated in the *Myd88*^KO^ group (Figure 3c(ii)). When tumor tissue was compared to non-treated bladder tissue samples, most of the differentially abundant genes were upregulated in both *Myd88*^KO^ and WT tumor groups (Figure 3c(iii,iv)).

Figure 3d illustrates differentially expressed genes across group comparisons. In non-treated bladder tissue, few genes draw attention. The metallothionein 1 and 2 genes (*Mt1* and *2*), which protect against oxidative stress and carcinogenesis [20], are four and five times upregulated in the *Myd88*^KO^ group (Figure 3c(i),d(i)). The angiopoietin-like 4 (*Angptl4*) gene, taking part in preventing metastasis [21,22], also has three times higher expression in *Myd88*^KO^ bladder samples (Figure 3c(i),d(i)). On the other hand, the nuclear receptor subfamily 4 group A member 1 gene (*Nr4a1*), which is an orphan receptor having a role in the induction of apoptosis [23], is four times downregulated in *Myd88*^KO^ mice compared to WT non-treated bladder tissue (Figure 3c(i),d(i)). Still, after tumor induction, this gene becomes five times more highly expressed in *Myd88*^KO^ mice compared to the WT group (Figure 3c(ii),d(ii)). In tumor samples, in addition to *Nr4a1*, several upregulated genes were pseudogenes (Figure 3c(ii),d(ii)). Furthermore, among downregulated genes, the C-X-C motif chemokine ligand 17 (*Cxcl17*) gene, which promotes tumorigenesis through its angiogenic activity [24], was 21 times less expressed in *Myd88*^KO^ samples (Figure 3c(ii),d(ii)). Other highly downregulated genes among *Myd88*^KO^ mice, such as *Myh1*, *Myl1*, and *Mylpf*, contribute to muscle contraction processes [25] (Figure 3c(ii),d(ii), Supplementary Table S2). Interestingly, when comparing tumor samples to non-treated controls, those genes contributing to muscle contraction processes that were downregulated in *Myd88*^KO^ tumor samples compared to WT tumor samples are now upregulated only in WT tumor samples compared to respective NT controls (Supplementary Table S4), while there are no differences between *Myd88*^KO^ tumor and NT samples (Supplementary Table S3). The matrix metalloproteinase 13 (*Mmp13*)gene, responsible for the breakage of the extracellular matrix and tumor cell invasion [26], is also three times downregulated in the *Myd88*^KO^ tumor group compared to WT (Supplementary Table S2). In a comparison of tumor samples to their respective non-treated controls, most of the up- and downregulated genes are present in both *Myd88*^KO^ and WT groups (Figure 3c(iii,iv),d(iii,iv)). The collagen type VII alpha 1 chain (*Col7a1*), known to promote neoplasia [27], was significantly upregulated, more than 8 and 15 times, in both *Myd88*^KO^ and WT tumor samples compared to their non-treated controls, respectively (Figure 3d(iii,iv)). On the other hand, the *Col7a1* gene was two times downregulated in *Myd88*^KO^ tumor samples compared to WT (Supplementary Table S5). Additionally, KEGG pathway analysis demonstrated that almost all genes associated with the “ribosome pathway” are downregulated in *Myd88*^KO^ tumor samples compared to WT tumors and *Myd88*^KO^ non-treated controls (Figure 3e). Although ribosomal protein-encoding genes were less than two times downregulated (Supplementary Table S6), all together they cause a significant change among *Myd88*^KO^ mice.

All differentially expressed genes (Supplementary Tables S1–S4) and gene ontology enrichment analysis (Supplementary Figure S3) are presented in the Supplementary Materials.

The inflammatory response was also assessed using the sequencing data. The Murine Microenvironment Cell Population counter (mMCP-counter), used to estimate the immune cell-type abundance scores, showed no differences between *Myd88*^KO^ and WT tumor and non-treated control bladder samples (*p* = 0.392) (Supplementary Figure S1c), suggesting inflammatory responses were not affected.

### 2.4. Proliferation and Apoptosis Profiling of Myd88^KO^ Bladder Tumors

**Figure 4:**
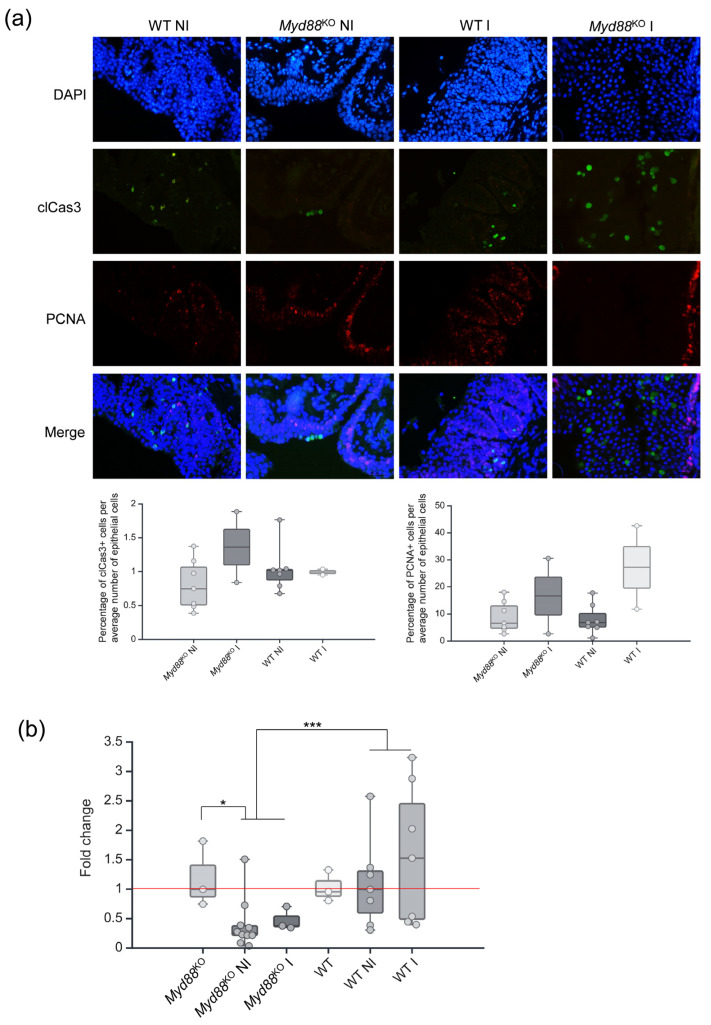
Molecular profiling of *Myd88*^KO^ bladder tumors. (**a**) Immunofluorescence analysis of clCas3 and PCNA expression in *Myd88*^KO^ and WT tumor samples (40× magnification); (**b**) qPCR data representing *Mmp13* gene expression in NI and I *Myd88*^KO^ and WT tumor samples with corresponding NT controls; DAPI—cell nuclei stained in blue; NI—non-invasive; I—invasive; * *p* < 0.05; *** *p* < 0.001; red line—baseline point at which there is no change.

To characterize cancer invasiveness, proliferation, and apoptosis status among *Myd88*^KO^ and WT tumors, proliferating cell nuclear antigen (PCNA) and cleaved caspase 3 (clCas3) were used as respective markers (Figure 4a). After randomly choosing three high-magnification fields per sample, representing NI and I tumors, the data were analyzed as a percentage of clCas3 and PCNA positive cells. The results showed no significant differences between the *Myd88*^KO^ and WT groups regarding apoptosis (*p* = 0.579) and proliferation (*p* = 0.475).

Next, we pursue the path of tumor cell migration and extracellular matrix remodeling. For that purpose, all NI and I samples from the *Myd88*^KO^ and WT groups from the chronic experiment and their respective three non-treated controls per group were tested for *Mmp13* expression using qPCR (Figure 4b). The obtained data showed that the baseline *Mmp13* expression in the *Myd88*^KO^ group was higher compared to *Myd88*^KO^ tumor samples (*p* = 0.022). Although there was a trend showing invasive tumors expressing more *Mmp13* than NI in both groups, the results were not significant. Comparing *Myd88*^KO^ and WT mice, a difference was detected when NI and I tumors were analyzed together, resulting in WT samples expressing more *Mmp13* (*p* < 0.001).

## 3. Discussion

Less invasive bladder tumors in mice lacking functional MyD88 signaling are the major finding of our study. As a key signaling transducer from most Toll-like receptors, MyD88 transmits signals about microbial presence and governs the appropriate cellular response [28]. The long-held postulate that the bladder and urine are sterile was disproved with the discovery of urinary microbiota [29]. Since then, there has been an increase in studies aimed at identifying the possible causes, triggers, or predictive variables in the roles played by the microbiota in the development of bladder cancer [11,30]. Since the influence of microbiota on bladder carcinogenesis is becoming more evident, there is a need for basic research reflecting the development of urinary bladder cancer in the context of nonfunctional microbial receptors and their signal transducers. In such a context, TLR4, a receptor that recognizes lipopolysaccharide from Gram-negative bacteria, and the signaling molecule MyD88 [16] are of primary importance.

Therefore, we challenged *Tlr4* and *Myd88* KO mice with a well-known urinary bladder carcinogen—BBN. To our surprise, *Tlr4*^KO^ mice did not show any difference in response to the carcinogen, either after acute or chronic exposure, while the *Myd88*^KO^ mice had a considerably lower prevalence of invasive tumors. The importance of MyD88 in the development of various cancers has been emphasized previously [28,31,32,33]. Most of the protumorigenic activities of MyD88 relate to the gain of function mutations [34]. The role of MyD88 in BC has not been directly tested thus far, although there are reported mutations of the *Myd88* gene in BC [35]. MyD88 signaling modulates various aspects of cancer progression, including tumor growth, invasion, metabolic reprogramming, and immune evasion [33]. Additionally, it plays a role in activating the Ras oncogenic pathway [36], which has been implicated in BC development [37,38]. Sun et al. demonstrated the impact of the circCEP128/miR-145-5p/MyD88 axis on promoting BC progression. More precisely, circCE28 is overexpressed in BC, leading to the subsequent downregulation of miR-145-5p and upregulation of MyD88 [39].

Given the absence of a functional receptor and a key signaling molecule mediating microbe–host interactions in the experimental mice, microbiota alterations and their influence on cancer development were expected. Notably, the *Myd88*^KO^ mice exhibited the lowest microbial diversity, a trend that persisted during tumor development. This downregulation of microbial diversity could influence the genes sensitive to colonization, such as *Angptl4* [40]. Furthermore, the genus *Faecalibaculum* had the highest abundance in *Myd88*^KO^ mice after the introduction of the carcinogen, a tendency that continued during the five-month experiment. The genus *Faecalibaculum* consists of butyrate-producing species with anti-tumor properties [41]. Butyrate, a short-chain fatty acid, is implicated in inhibiting carcinogenesis across various cancer types, such as colorectal [42], breast [43], and bladder [44]. Its effects on bladder cancer have been demonstrated in both mouse models and cell lines [44,45,46,47]. Butyrate, a histone deacetylase inhibitor, exerts its anti-tumor effects by promoting apoptosis and suppressing proliferation, and migration [46].

Since differences in the expression of proliferative PCNA and apoptotic clCas3 markers were not observed, another possibility was that the invasion of cancer cells in the *Myd88*^KO^ group was affected. RNA sequencing results indicated that the *Myd88*^KO^ samples exhibited decreased expression of the matrix metalloproteinase 13 (*Mmp13)* gene in NI tumors compared to WT samples, while qPCR analysis revealed differences when NI and I samples were pooled, confirming higher *Mmp13* expression in the WT mice tumors. Matrix metalloproteinase 13 is a collagenase associated with the degradation of the extracellular matrix (ECM) and its overexpression is seen in several cancers, including BC; it leads to enhanced tumor progression, migration, and metastases [26]. *Mmp13* gene downregulation in the *Myd88*^KO^ tumor group could be attributed to the fact that the MMP13 protumor activity may be mediated through the MyD88/ERK/NF-κB signaling pathway [48,49,50]. Furthermore, protective gene expression patterns seen in *Myd88*^KO^ mice, including the *Mt1*, *Mt2*, *Angptl4*, *Nr4a1*, *Cxcl17*, *Mmp13*, *Myh1*, *Myl1*, and *Col7a1* genes, may synergistically suppress BC development and progression [20,22,24,25,27].

In conclusion, the finding that *Myd88*^KO^ mice have changed microbiota and less invasive bladder tumors underscores the importance of both the microbiota and the MyD88 signaling pathway in the development of BC. Our results suggest that modulation of MyD88 signaling and microbiota composition in clinical settings could reduce BC invasiveness.

## 4. Materials and Methods

### 4.1. Animal work

*Tlr4*^KO^ (B6(Cg)-Tlr4tm1.2Karp/J) and *Myd88*^KO^ (B6.129P2(SJL)-Myd88tm1.1Defr/J) mouse models and associated wild type (WT) B6 controls were purchased from The Jackson Laboratory. All mice were inbred animals of the C57BL/6J strain. The mice used in the experiments were males, 6–8 weeks old. Standard husbandry conditions included 20 to 24 °C temperatures, 50 ± 20% humidity, light and dark cycles of 12 h each, and certified wooden litter (Mucedola srl, Settimo Milanese, Italy). Food and water (autoclaved) were available to the animals *ad libitum*.

### 4.2. BBN Mice Model

*Myd88*^KO^ (n = 37 acute and n = 15 chronic experiment), *Tlr4*^KO^ (n = 21 acute and n = 16 chronic), and WT (n = 49 acute and n = 48 chronic) mice were challenged with 0.05% N-butyl-N-(4-hydroxylbutyl)-nitrosamine (BBN) in drinking water during two (acute) and twelve weeks (chronic protocol). After 12 weeks of consuming BBN, all mice were administered autoclaved drinking water without BBN, making it a 20-week chronic experiment (Figure 1A). Before the onset of the chronic treatment, feces from the colon (pellets) were collected from all mice in each group for 16S metagenomic sequencing. Pellets were also collected from all mice undergoing chronic treatment after 12 weeks of BBN treatment (Figure 2A). At the end of the treatment, 2 weeks for acute and 20 weeks for chronic experiment, all mice groups were sacrificed. Urinary bladder tissue, as well as feces from the caecum and colon of mice undergoing chronic experiments, was collected. All fecal samples were immediately frozen and urinary bladders were cut medially into two halves. One half was frozen for the following molecular analyses and the other half was washed in PBS and immersed in 4% paraformaldehyde (PFA) (Sigma-Aldrich, Germany) for histological analysis.

### 4.3. Histological Analysis

After 24 h incubating in 4% PFA, samples were dehydrated with an ascending series of ethanol dilutions, followed by clearing in xylene and embedding in third paraffin. Paraffin-embedded tissue was cut into four-micron-thick sections and stained with hematoxylin (Sigma-Aldrich, Darmstadt, Germany) and eosin (Merck, Darmstadt, Germany). Sample images were captured using an Olympus BX43 microscope (Olympus, Tokyo, Japan) at 4× and 40× magnifications, and an experienced pathologist, who was blind for the sample group affiliation, performed pathohistological analysis.

### 4.4. DNA Isolation

DNeasy PowerSoil Pro Kit (Qiagen, Hilden, Germany) was used for microbial genomic DNA extraction from fecal samples, following the manufacturer’s protocol. The quantity and purity of extracted DNA were measured with a NanoDrop 1000 spectrophotometer (ThermoFisher Scientific, Waltham, MA, USA).

### 4.5. 16S Metagenomic Sequencing

Genomic DNA sample quality control (QC), amplification of the bacterial 16S V4 region (300 bp), amplicon library preparation, sequencing, and data QC were performed by Novogene (Beijing, China). Paired-end sequencing of 250 bp was conducted using the Illumina NovaSeq 6000 (Illumina, San Diego, CA, USA) platform with 30 K tags of raw data per sample. Raw sequences obtained during 16S metagenomic sequencing were submitted to the NCBI Sequence Read Archive (accession number PRJNA1119950).

### 4.6. Total RNA Isolation

Total RNA was isolated from all *Myd88*^KO^ and WT frozen urinary bladder tissue samples from chronic experiments, as well as from their *Myd88*^KO^ and WT non-treated controls (3 in each group), using the TRIzol reagent (Invitrogen, Waltham, MA, USA). Samples were homogenized with Minilys homogenizer (Bertin, Montigny-le-Bretonneux, France) and the downstream RNA isolation was performed according to the manufacturer’s protocol. The quantity and purity of extracted RNA were measured with a NanoDrop 1000 spectrophotometer (ThermoFisher Scientific, Waltham, MA, USA). Genomic DNA contamination and RNA quality were assessed through agarose gel electrophoresis and with the Agilent 5400 (Agilent Technologies, Santa Clara, CA, USA) (integrity number > 8).

### 4.7. RNA Sequencing

RNA sequencing was performed at Novogene (Cambridge, UK). The total RNA from 3 NI (non-invasive) tumor samples from *Myd88*^KO^ and WT groups, as well as 3 samples from each *Myd88*^KO^ and WT non-treated group, was analyzed. Following mRNA purification from total RNA samples using poly-T oligo-attached magnetic beads, a cDNA was synthesized, and the library was constructed. Quantification of the libraries was conducted with Qubit and real-time PCR, and a bioanalyzer was used for size distribution detection. Quantified libraries were pooled and the paired-end 150 bp sequencing was performed on the Illumina NovaSeq 6000 (Illumina, San Diego, CA, USA) platform, obtaining at least 12 G of raw data per sample. Raw sequences obtained during RNA sequencing were submitted to the NCBI Sequence Read Archive (accession number PRJNA1119950).

### 4.8. Immunofluorescence Analysis

Four-micron-thick slides of paraffin-embedded *Myd88*^KO^ and WT BC tissue were deparaffinized with xylene, and rehydration was performed with a descending series of alcohol concentrations. EDTA buffer (pH = 8.0) was used for antigen retrieval. Anti-clCas3 rabbit (Asp 175, Cell Signaling Technology, Danvers, MA, USA) and anti-PCNA mouse (PC-10) (sc56, Santa Cruz Biotechnology, Dallas, TX, USA) primary antibodies were incubated overnight at 4 °C at a dilution of 1:100 and 1:200, respectively. After washing in PBS, secondary antibodies, Alexa fluor 488 anti-rabbit (A21206, Invitrogen, Waltham, MA, USA) and Alexa fluor 568 anti-mouse (A11004, Invitrogen, Waltham, MA, USA) at a dilution of 1:1000 were incubated for one hour. DAPI (40,6-diamidino-2-phenylindole) was applied for one minute and washed in PBS, after which, mounting media was applied and the slides covered. Samples were recorded at 40x magnification using the Olympus BX43 microscope (Olympus, Tokyo, Japan). The Fiji program [51] was used for photo processing and analysis.

### 4.9. cDNA Synthesis

Complementary DNA (cDNA) was synthesized from 1 μg of RNA (*Myd88*^KO^ and WT tumor samples) in 20 μL of reaction mix per sample using the High-Capacity cDNA Reverse Transcription Kits (Applied Biosystems, Foster City, CA, USA), following the manufacturer’s protocol.

### 4.10. qPCR

Quantitative PCR (qPCR) analysis of matrix metalloproteinase 13 (*Mmp13*) expression was performed in all tumor samples from *Myd88*^KO^ and WT mice, with respected non-treated controls (3 per group), using the SsoAdvanced Universal SYBR Green Supermix (Bio-Rad, Hercules, CA, USA) on the CFX96 Touch Real-Time PCR Detection System (Bio-Rad, Hercules, CA, USA) following the manufacturer’s protocol. All reactions were performed in duplicate. Each run included the ribosomal protein S23 (*Rps23*) as a reference gene control. Mouse *Mmp13* and *Rps23* primers were designed using the NCBI Primer-BLAST tool (Table 1).

### 4.11. Statistical Analysis

Statistical analysis was performed in R version 4.2.2 [52]. Fisher’s exact test was used to assess the differences between the *Myd88*^KO^, *Tlr4*^KO^, and WT groups regarding degenerative changes in acute experiments and pathohistological tumor staging in chronic experiments, as well as to assess differences in inflammatory scores between the *Myd88*^KO^ and WT groups in acute and chronic experiments.

After obtaining 16S sequencing raw data, the Qiime2 platform [53] was used to perform denoising with Dada2 [54], generate an ASV feature table, assign taxonomy (Silva 138), perform rarefaction, and calculate alpha (observed features, Faith_PD, and Shannon) and beta diversity (Bray–Curtis and weighted UniFrac) measures. Alpha diversity differences were calculated using the Kruskal–Wallis (pairwise) test, and the pairwise Permanova test was used to determine differences regarding beta diversity. Beta diversity measures were visualized using the PCoA Emperor plots [55]. Differential abundance analysis was performed using the ANCOM-BC2 package [56].

The Hisat2 v2.0.5 alignment program was used to map raw RNA-seq reads to the mouse reference genome (Genome Assembly GRCm39 GCA_000001635.9). Quantification of the read numbers mapped to each gene was performed using the FeatureCounts algorithm v1.5.0-p3. Gene expression levels were expressed as FPKM (fragments per kilobase of transcript sequence per million base pairs sequenced). Adjusted *p*-value < 0.05 and |log2 fold change| > 1 thresholds were set to define differentially expressed genes (DEGs). DESeq2 package v1.40.2 [57] was used to perform the differential gene expression analysis using the recommended default parameters. The Murine Microenvironment Cell Population counter (mMCP-counter) [58], within the immunedeconv package [59], was used to estimate the cell-type abundance scores in *Myd88*^KO^ and WT tumor and non-treated control bladder samples.

The Kruskal–Wallis test with post-hoc Dunn’s test was used to assess the differences in clCas3 and PCNA expression between *Myd88*^KO^ and WT tumor samples, as well as to analyze inflammation data obtained from the mMCP-counter. RT-PCR data analysis of *Mmp13* gene expression in tumor samples was performed using the Mann–Whitney U test.

Statistical significance for all tests was set at *p* < 0.05 and presented as follows: ns—nonsignificant, * *p* < 0.05, ** *p* < 0.01, and *** *p* < 0.001.

## Figures and Tables

**Table 1:** Primers used for qPCR analysis.

Gene	Forward Primer	Reverse Primer
m*Mmp13*	5′ CCACTCCCTAGGTCTGGATCA 3′	5′ CTTCATCGCCTGGACCATAA 3′
m*Rps23*	5′ AATGCCTTGTGGGTCCTTCC 3′	5′ CACGACACTTGCCCATCTTG 3′

## Data Availability

The original contributions presented in the study are included in the article and Supplementary Materials; further inquiries can be directed to the corresponding author.

## Supplementary Materials

The following supporting information can be downloaded at: https://www.mdpi.com/article/10.3390/ijms25137176/s1.
