# Supplementary material for: MyD88 Signaling Accompanied by Microbiota Changes Supports Urinary Bladder Carcinogenesis
Source: Int J Mol Sci. 2024 Jun 29;25(13):7176. doi: 10.3390/ijms25137176 (PMC11241070; doi:10.3390/ijms25137176)
Supplement: Supplementary file 1 [file ijms-25-07176-s001.zip › Supplementary figures.pdf]

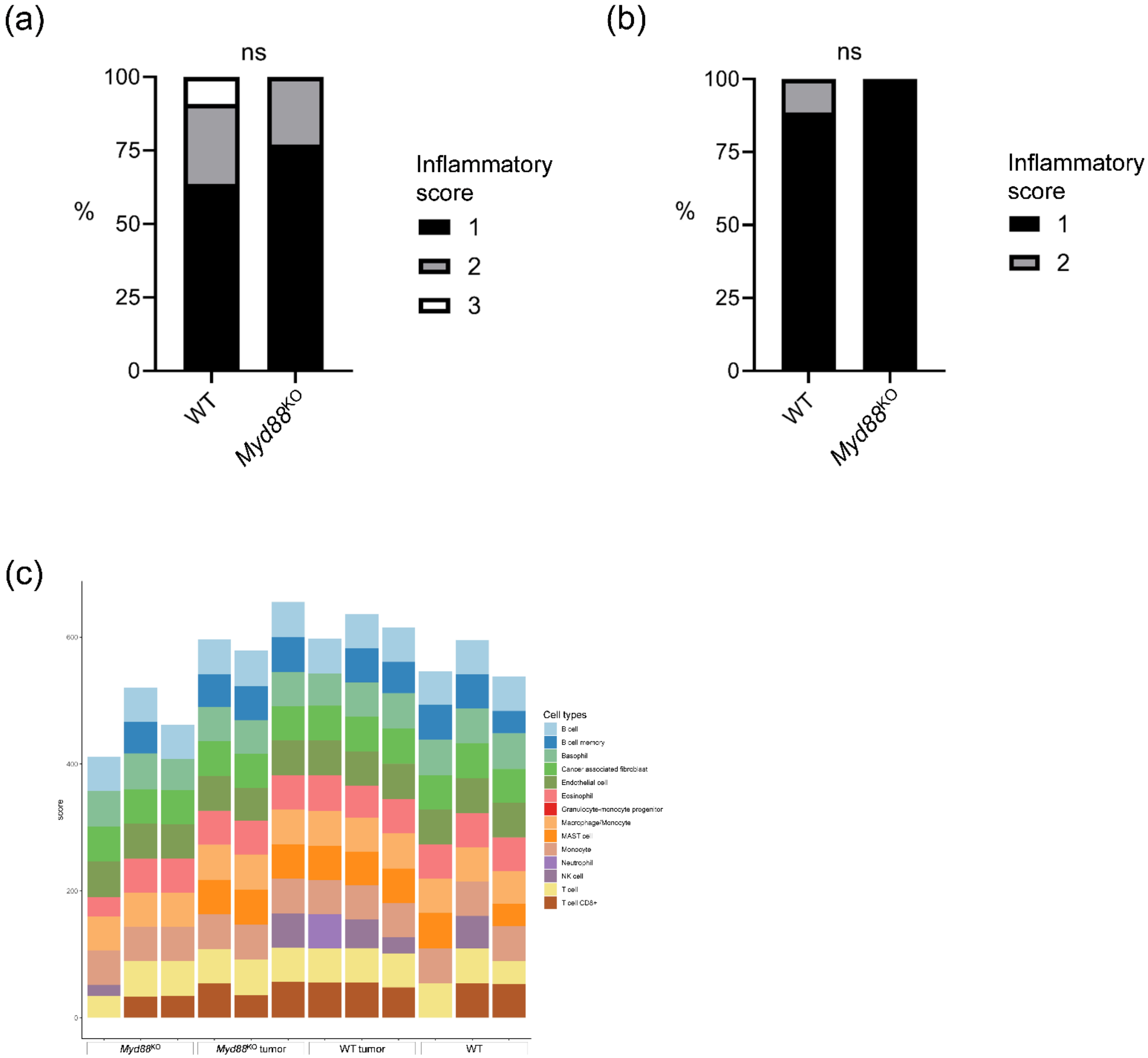

Figure S1: Assessment of inflammatory response during acute and chronic experiments in *Myd88*^{KO} and WT groups. (**a**) Evaluation of inflammatory scores in *Myd88*^{KO} and WT tumor groups in the acute experiment; (**b**) Evaluation of inflammatory scores in *Myd88*^{KO} and WT tumor groups in the chronic experiment; (**c**) Murine Microenvironment Cell Population counter (mMCP-counter) estimation of the cell-type abundance scores in *Myd88*^{KO} and WT NI tumor and non-treated control bladder samples.

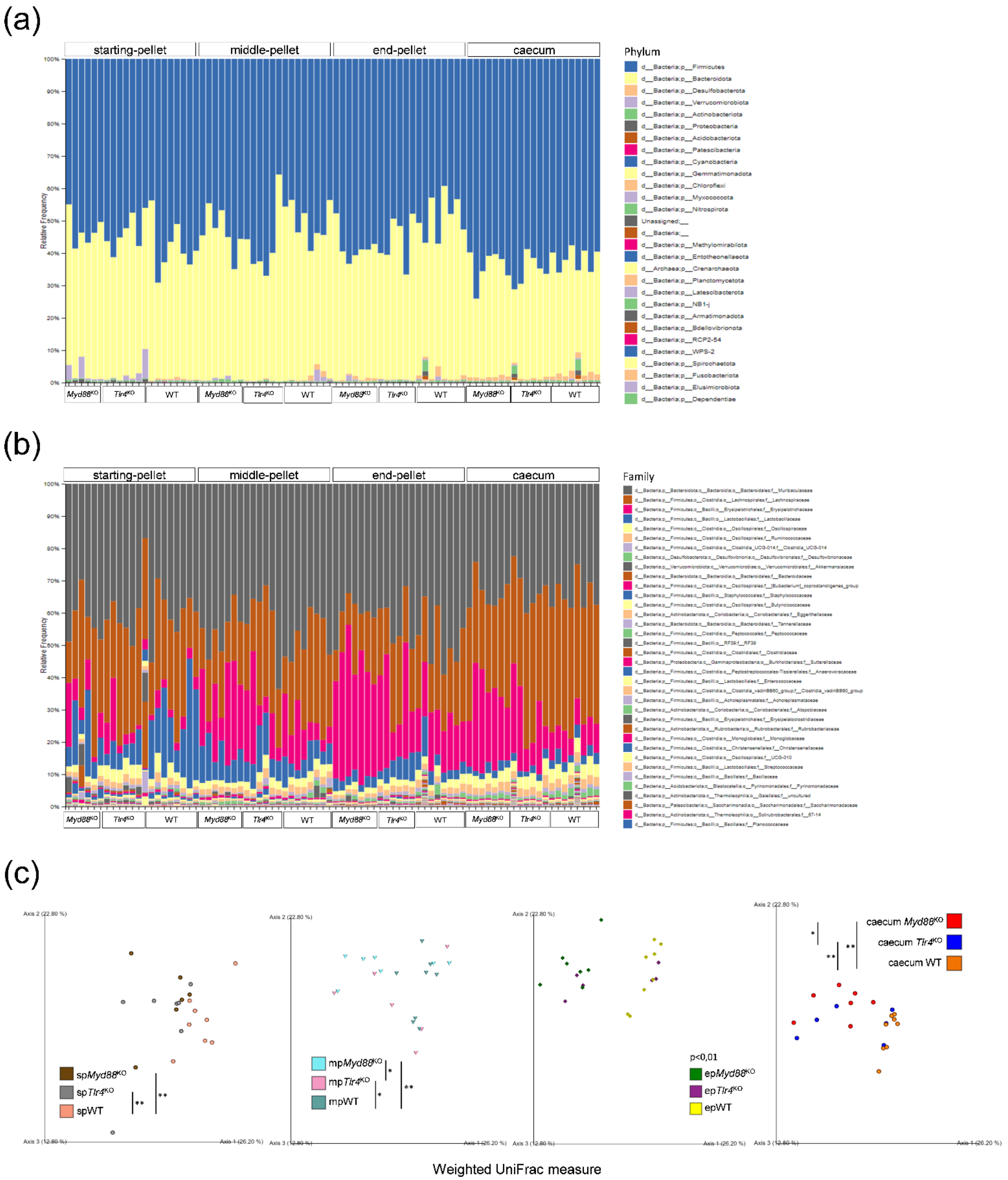

Figure S2: 16S metagenomic analysis of stool samples from the chronic experiment. (**a**) Taxonomy bar plot for phylum taxonomic level considering time points and mice groups; (**b**) Taxonomy bar plot for family taxonomic level considering time points and mice groups; (**c**) PCoA plots representing Weighted UniFrac beta diversity measure considering time points and mice groups. sp – starting pellet (before the BBN treatment); mp – middle pellet (after 12 weeks of BBN treatment); ep – end pellet (at the end of the chronic experiment after 20 weeks; * p < 0.05; ** p < 0.01.

Myd88^{KO} vs. WT

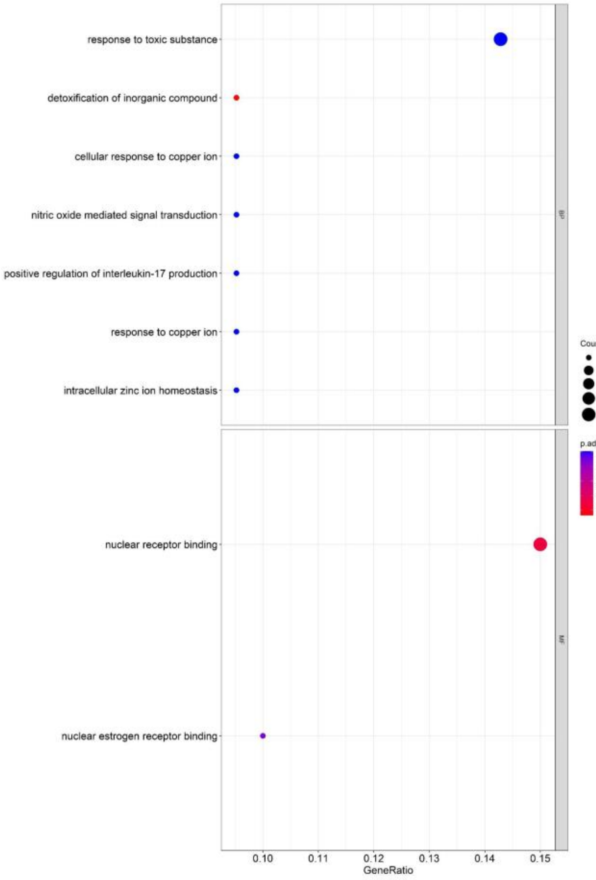

Myd88^{KO} tumor vs. WT tumor

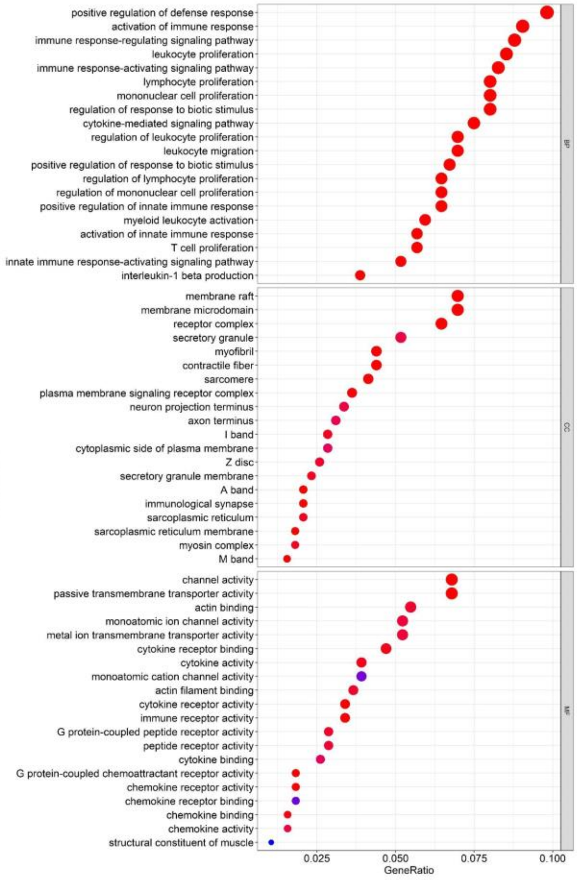

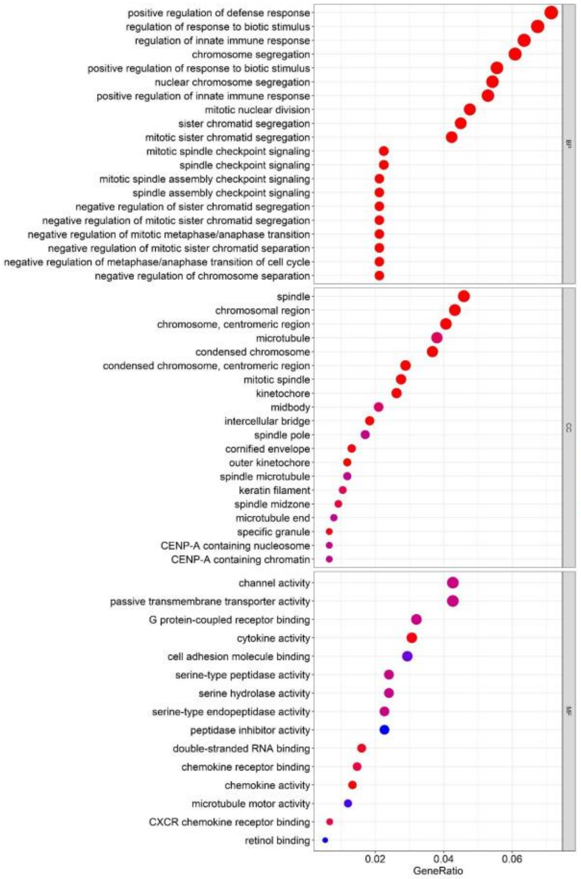

Myd88^{KO} tumor vs. *Myd88*^{KO}

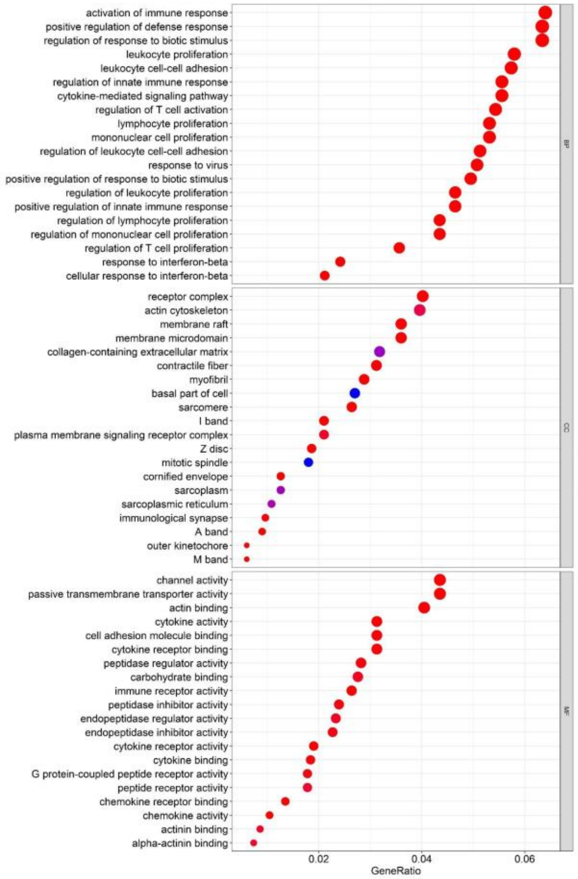

WT tumor vs. WT

Figure S3: Gene Ontology (GO) analysis. BP – biological processes; CC – cell signaling; MF – molecular functions.
